# Long-term priority effects among insects and fungi colonizing decaying wood

**DOI:** 10.1111/j.1365-2656.2011.01860.x

**Published:** 2011-11

**Authors:** Jan Weslien, Line B Djupström, Martin Schroeder, Olof Widenfalk

**Affiliations:** 1 The Forestry Research Institute of Sweden (Skogforsk)SE-75183, Uppsala; 2 Department of Ecology, Swedish University of Agricultural SciencesBox 7044, SE-75007, Uppsala

**Keywords:** biodiversity, competition, coarse woody debris, facilitation, saproxylic, stochastic, succession

## Abstract

**1.** Priority effects have been hypothesized to have long-lasting impact on community structure in natural ecosystems. Long-term studies of priority effects in natural ecosystems are however sparse, especially in terrestrial ecosystems.

**2.** Wood decay is a slow process involving a high diversity of insect and fungus species. Species interactions that drive change in communities of insects and fungi during wood decay are poorly understood because of a lack of sufficient long-term studies.

**3.** In this paper, we followed the colonization and succession of wood-living insects and fungi on cut trees during 15 years, from tree death and onwards, in a boreal forest landscape. We test the long-term priority effects hypothesis that the identity and abundance of species that colonize first affect the colonization success of later-arriving species. We also hypothesize that species interact in both facilitative and inhibitory ways, which ultimately affect habitat quality for a red-listed late-succession beetle species.

**4.** Possible causal associations between species were explored by path analysis. The results indicate that one bark beetle species, *Hylurgops palliatus*, and one wood-borer species, *Monochamus sutor*, which colonized the wood during the first year after cutting, influenced the occurrence of a rare, wood-living beetle, *Peltis grossa*, that started to emerge from the stumps about 10 years later. The positive effects of *Hylurgops palliatus* and negative effects of *M. sutor* were largely mediated through the wood-decaying fungus species *Fomitopsis pinicola*.

**5.** The study shows that variable priority effects may have long-lasting impact on community assembly in decaying wood. The study also exemplifies new possibilities for managing populations of threatened species by exploring links between early, well-understood species guilds and late, more poorly understood species guilds.

## Introduction

A controversial subject in ecology concerns the forces that drive the formation of species assemblages and whether communities are formed principally by deterministic processes coupled to the environment or by stochastic processes coupled to random invasions and extinctions (Hubbel 2001; Chase 2003; Nee & Stone 2003; Lortie *et al.* 2004; Ejrnæs, Bruun & Graae 2006; Swenson, Anglada-Cordero & Barone 2011). An alternative hypothesis proposes that interactions between deterministic and stochastic processes are coupled to stochastic variation in colonization history (Chase 2010), and many studies have suggested that community assemblages are influenced by the sequence of colonizations and the outcomes of past species interactions (Diamond 1975; Drake 1991; Fukami *et al.* 2007, 2010; Olito & Fukami 2009; Chase 2010). Thus, the identity and abundance of species that colonize first may affect the colonization success of later-arriving species. Such priority effects may be positive (facilitative) or negative (inhibitory) (Connell & Slatyer 1977). Chase (2010) argued that priority effects probably explain much of the variation found in community structures across different localities, with similar environmental conditions.

Priority effects have been shown to have a profound influence on species assemblages in coral reefs (Shulman *et al.* 1983; Almany 2003) and ponds (Alford & Wilbur 1985; Warner, Dunson & Travis 1991; Chase 2003, 2010). Most studies in terrestrial systems have however been in microcosms on a relatively small scale (Shorrocks & Bingley 1994; Kennedy & Bruns 2005; Ejrnæs, Bruun & Graae 2006; Fukami *et al.* 2010) or of short-term field studies (Ehmann & MacMahon 1996; Viktorsson 2009). Long-term field studies, especially in forest ecosystems, are lacking.

Succession during decomposition of an ephemeral resource, such as a patch of wood, carrion or dung, so-called heterotrophic succession, is characterized by a particular sequence of species arrival. Decaying wood is typified by a particular sequence of insect and fungus species, many of which are broadly associated with a certain decay stage (Saalas 1917; Palm 1951; Swift 1977; Rayner & Boddy 1988; Boddy 2001; Ehnström & Axelsson 2002). The first species to colonize a newly dead or dying tree are beetles that feed on the nutrient-rich phloem and fungi that colonize broken or cut stems or scars in the bark. The sequence of arrival is best known among these pioneer species because they include those that kill trees or destroy timber with commercial value. Nevertheless, wood may take decades to decay completely, during which time the community structure of insects and fungi change as members undergo complex interactions with each other. Such patterns of interactions are only poorly understood because no study lasting a sufficiently long time has included both insects and fungi.

Both facilitative and inhibitory interactions are likely to occur between species inhabiting dead trees. Interspecific competition occurs both among insects (e.g. Schroeder & Weslien 1994) and fungi (e.g. Boddy 2000). Insects may disperse fungi and so facilitate their colonization (Rayner & Boddy 1988; Boddy & Jones 2008; Persson, Ihrmark & Stenlid 2011), and fungal mycelia are an important food sources for many insect species (Wilding *et al.* 1989). Several studies indicate that the insect community in decaying wood largely is formed by the type of rot in the wood, which is highly dependent on fungus species (Saalas 1917; Palm 1951, 1959; Jonsell, Schroeder & Weslien 2005).

Insect and fungus species dependent on dead trees in boreal forest ecosystems form a large group among which are included many endangered species (Dahlberg & Stokland 2004; Tikkanen *et al.* 2006; Gärdenfors 2010). It is therefore essential to better understand how habitats emerge and develop in dead trees to preserve biodiversity in general and to protect these threatened and endangered species in particular.

In this paper, we present results from a 15-year study, during which we followed the colonization and succession of wood-living insects and fungi. We test the long-term priority effects hypothesis that the identity and abundance of species that colonize first affect the colonization success of later-arriving species. We also hypothesize that species interact in both facilitative and inhibitory ways, which ultimately affect habitat quality for a particular species of wood-beetle currently listed as ‘vulnerable’ in a boreal forest ecosystem in Sweden.

## Materials and methods

### The system

The study system includes five beetle and two fungus species living on Norway spruce, *Picea abies* L. Karst. Four of the beetle species, the scolytids *Ips typographus* (L.), *Pityogenes chalcographus* (L.) and *Hylurgops palliatus* (Gyll.) and the cerambycid *Monochamus sutor* (L.), are among the most common early colonizers on dying or newly dead spruce in Northern Europe. The arrival sequence and approximate time for flight initiation in central Sweden are as follows: (i) *H. palliatus* in mid-April, (ii) *I. typographus* and *P. chalcographus* in mid-May and (iii) *M. sutor* in mid-June (Eidmann & Klingström 1990). The fifth beetle species, *Peltis grossa* (L.) (Trogossitidae), is rare and arrives several years after the other species. It is known to breed in standing stems with brown-rotted wood of different tree species (Saalas 1917; Palm 1951). A positive association between the bracket fungi *Fomitopsis pinicola* (Sw.) P. Karst and *P. grossa* has been proposed previously (Ehnström & Axelsson 2002; Krasutskii 2007). Because of the lack of suitable breeding substrate in today's managed forests, *P. grossa* has become rare (Fjellberg & Hansen 1997; Ehnström 2001) and is now classified as ‘vulnerable’ in the Swedish Red List according to the IUCN criteria (Gärdenfors 2010). The adult *P. grossa* leaves a characteristic oval emergence hole about 5 × 10 mm (Ehnström & Axelsson 2002).

The bracket fungi *F. pinicola* and *Trichaptum abietinum* (Dicks.: Fr.) Ryv. are very common species and among the first fungi to colonize dead tree trunks (Käärik & Rennerfelt 1957; Lindhe, Åsenblad & Toresson 2004). Insect communities in wood rotted by *T. abietinum* and wood rotted by of *F. pinicola* differ in their assemblage (Jonsell, Schroeder & Weslien 2005). *Fomitopsis pinicola* causes brown-rot and *T. abietinum* white-rot, and the two species grow spatially separated (Renvall 1995; Abrahamsson, Lindbladh & Rönnberg 2008). The fruiting bodies of *F. pinicola* are perennial and sporulate from early spring to autumn (Nuss 1986), whereas the fruiting bodies of *T. abietinum* are annual and start to sporulate later during the growing season. Both species may be dispersed by wind and by bark beetles (Pettey & Shaw 1986, Persson *et al.* 2009; Persson, Ihrmark & Stenlid 2011). *Fomitopsis pinicola* can occasionally live as a parasite on living trees of decreased vitality (Forestry Compendium, 2005 and references therein). In the present study, only vigorous trees were felled (see below), and thus the possibility for them to be pre-infected with the fungus is very unlikely.

### Field study

To study the sequence in which beetles and fungi colonize standing, coarse dead-wood, high stumps of Norway spruce were created in 1994 and 1995 near Grangärde in central Sweden (60°16′00″N; 14°59′00″E). Prior to conventional clear cutting of six managed spruce stands, 45–100 dominant, apparently healthy spruce trees in each stand were selected to be high-cut. Trees were cut 1·3–5·3 m (mean = 2·8, SD = 0·7) above ground, and the remaining stump was left rooted in the ground. Diameter at breast height (1·3 m above ground level) ranged from 18 to 59 cm (mean = 34·7, SD = 7·2). The total number of high-cut stumps was 425, of which 363 were followed during the entire study period. Cutting time varied between sites and within two of the sites, giving three cohorts of stumps (Table 1). Distances between sites ranged from 3 km to 5 km except for two sites (D and E), which were only 0·7 km apart. On all sites except site D, most stumps were exposed to the sun during the warmest part of summer days (south and west). At site D, stumps were situated along a shaded forest edge facing northeast. More detailed information is given in Schroeder *et al.* (1999).

**Table 1:** Number of stumps by site and cohort

	Cutting season/first summer after cutting			
		
Site	Winter/1994	Autumn/1995	Winter/1995	Total
A	87	0	0	87
B	75	0	0	75
C	78	0	0	78
D	0	19	26	45
E	0	18	24	42
F	0	36	0	36
Total	240	73	50	363

Stumps in the 1994 cohort were inspected in autumn 1994 and thereafter stumps in all cohorts were inspected in autumn 1995, 1997, 1998 and each year from 2003 to 2009. The areas of bark with galleries of *H. palliatus*, *P. chalcographus* and *I. typographus* were recorded as percentages in the year of felling (1994 or 1995) and transformed to absolute areas of colonized bark. The three species together covered an average of 59% of the bark area. An average of 30% of the bark area was not colonized by any bark beetle species during the first summer. The remaining c. 10% of the total bark area was occupied by *Orthotomicus* spp (3 possible species), which were not included in the analysis as these bark beetles only could be identified to genus. The reason for this was that their galleries are not very species-characteristic and the method used for estimating area of bark colonized by bark beetles had to be non-destructive, i.e. the bark was not peeled off. A more detailed description of the method, abundance of different bark beetle species and relationships between bark beetle species are given in Schroeder *et al.* (1999). Emergence holes of *M. sutor* were recorded in 1997 (3rd or 4th year after felling). The number of emergence holes of *P. grossa*, the percentage of stump surface area covered by bark, the number of living and dead fruiting bodies of *F. pinicola* and the occurrence of *T. abietinum* were recorded at each inspection. *Trichaptum abietinum* occurrence was assigned to one of three classes based on the frequency of fruiting bodies: absent, 0; < 25% coverage of stump surface area, 1; ≥ 25% coverage, 2.

The main start of *P. grossa* emergence was in 2003. This conclusion is based on field observations in July and September 2003. On a sample of 222 stumps, we found nine emergence holes on six stumps in July. All emergence holes except one were judged to be made the current summer. In September 2003, the number of emergence holes on the same 222 stumps had increased to 81 on 13 stumps.

### Statistical analysis

**Fig. 1:**
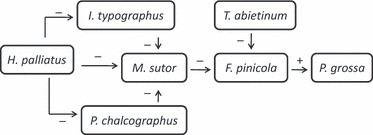
Hypothetical relationships between species. *Hylurgops palliatus*, the earliest colonizer, reduces habitat availability for *Ips typographus*, *Pityogenes chalcographus* and *Monochamus sutor*. *Ips typographus* and *P. chalcographus*, which fly at the same time, may reduce habitat availability for the later-arriving *M. sutor.* Field observations indicated that stumps with signs of *M. sutor* lost their bark early, which was seemingly negative for *Fomitopsis pinicola*. *Trichaptum abietinum* causes white-rot and *F. pinicola* causes brown-rot, which is a prerequisite for *Peltis grossa*. Direct associations are indicated with arrows, and negative or positive associations are indicated with signs.

A hypothetical model of causal species associations was tested (Fig. 1). The strongest organizing factor in this model is the arrival time of species. For instance, because *H. palliatus* arrives first, all paths connected to it have no alternative other than leading from it; similarly because *P. gross*a arrives last, all paths connected to it must lead towards it. The model was tested in two steps: first, the effects of 10 quantitative and categorical predictor variables on the number of *P. grossa* emergence holes (the latest colonizer) were analysed in general linear mixed models, PROCMIXED (SAS-inst. 2003); second, the continuous variables that had strong effects (*P* < 0·001) were included in a path analysis in which possible causal links between variables were analysed.

**Fig. 2:**
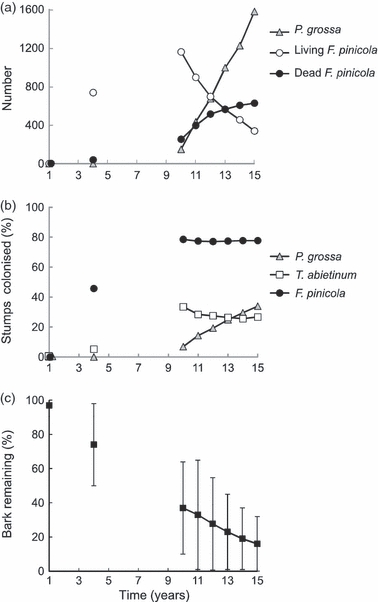
Development of 363 high stumps over time. (a) Total number of *Peltis grossa* emergence holes and living and dead fruiting bodies of *Fomitopsis pinicola.* (b) Proportion of stumps colonized by *P. grossa, F. pinicola* and *Trichaptum abietinum.* (c) Proportion remaining bark ± Standard Error (SE).

The response variable in the PROCMIXED analyses was ‘ln (1 + cumulative number of *P. grossa* emergence holes per stump in year 15)’. The log-transformation was made to achieve normality and avoid over-dispersion in the data. Each of the predictor variables was tested separately and the model controlled for random effects of site (categorical). The predictor variables for each stump were: (i) ln (1 + cumulative number of living and dead *F. pinicola* fruiting bodies in year 10); (ii) frequency class of *T. abietinum* in year 10; (iii) ln (1 + cumulative number of *M. sutor* emergence holes in year 3 or 4); (iv) area of bark colonized by *I. typographus* in year 1; (v) area of bark colonized by *P. chalcographus* in year 1; (vi) area of bark colonized by *H. palliatus* in year 1; (vii) ln (stump diameter); (viii) ln (stump height); (xi) cutting year (categorical); and (x) cutting season (categorical). For the early colonizing beetles, there was no choice of which year after cutting to use in the analyses. Estimates of variables relating to the two fungi and *P. grossa* were drawn from the data pertaining to the year that was thought might give the best possible estimate, i.e. a year in which numbers had increased since the previous inspection but did not increase later (year 10 for *T. abietinum* and *F. pinicola*, year 15 for *P. grossa*, see Fig. 2).

The path analyses of causal covariance structures were performed in the CALIS procedure in SAS, which provides goodness-of-fit tests for the whole path model using iterative maximum likelihood methods. Apart from the classical Chi-square statistics, the CALIS procedure also provides a goodness-of-fit index (GFI), (Jöreskog & Sörbom 1985), which should be between zero and one. If GFI is negative or larger than one, the data probably do not fit the model. It is not possible to include categorical variables in path analysis (Petraitis, Dunham & Niewiarowski 1996); therefore, because the PROCMIXED analysis controlled for site effects, a site was excluded from the path analysis if any of the species was absent at that site. This was the case for site D where no emergence holes of *M. sutor* were found. We tested the path analysis also with site D included and, it produced similar results, the only differences being that the associations between *M. sutor* and other species became somewhat weaker and that the unexplained variance for *F. pinicola* and *P. grossa* became somewhat higher as compared to when site D was omitted.

## Results

The number of living fruiting bodies of *F. pinicola* peaked between years 4 and 10 after which the number of dead fruiting bodies increased (Fig. 2a). The proportion of stumps with *F. pinicola* present increased from 0 to 50% during the first 4 years after cutting and peaked at *c.* 80% between years 4 and 10. The pattern for *T. abietinum* was similar but with lower frequency (Fig. 2b). No fruiting bodies of either species appeared year 1. In year 2, four stumps were found with *F. pinicola* and two with *T. abietinum* (1994 cohort, 1995 assessment, data not shown). The total number of *P. grossa* emergence holes increased linearly from year 10 (Fig. 2a), with the mean number per stump in year 15 varying from 3·1 to 6·9 at all sites except site D, which had a mean of only 0·6. In year 15, the proportion of stumps with *P. grossa* emergence holes varied over the six sites from 23% (site D) to 57% (site F) with an overall average of 36%, and 16% of stumps with more than 10 emergence holes. Bark coverage decreased linearly over time to an average of less than 40% by year 10 (Fig. 2c).

## Species associations

The PROCMIXED analysis revealed *F. pinicola, M. sutor*, *T. abietinum, H. palliatus,* stump diameter and stump height to have strong effects on numbers of *P. grossa* (Table 2). The effects of *M. sutor* and *T. abietinum* were negative; those of all others were positive.

**Table 2:** Simple PROCMIXED models controlled for random effects of site. Each predictor variable was tested separately against the response variable. Ln(*x* + 1)-transformed values for the response variable *Peltis grossa* (emergence holes per stump) and the predictor variables *Fomitopsis pinicola* (fruiting bodies per stump), *Monochamus sutor* (emergence holes per stump). Ln-transformed values for stump diameter and stump height. Untransformed values for *Trichaptum abietinum* frequency and area of bark colonized by *Ips typographus, Pityogenes chalcographus* and *Hylurgops palliatus*

	Response variable *P. grossa*			
	
Predictor variable	Coeff.	SE	*t-*value	*P*-value
*F. pinicola*	0·76	0·06	12·5	<0. 0001
*T. abietinum*	−0·32	0·09	−3·7	0·0003
*M. sutor*	−0·38	0·07	−5·2	<0·0001
*I. typographus*	0·10	0·05	2·1	0·04
*P. chalcographus*	0·062	0·08	0·8	0·4
*H. palliatus*	0·28	0·084	3·6	0·0003
Stump diameter	1·45	0·30	4·8	<0·0001
Stump height	1·17	0·26	4·4	<0·0001
Year				
1994	−0·11	0·31	−0·34	0·7
1995	–	–		–
Cutting season				
Autumn	0·074	0·21	0·35	0·7
Winter	–	–		–

**Fig. 3:**
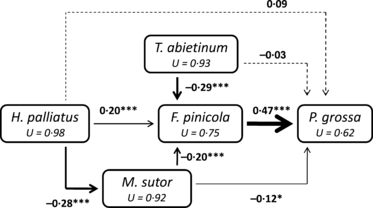
Suggested pathways during 15 years of wood decay, from *Hylurgops palliatus* colonization to *Peltis grossa* emergence. Coefficients indicate direct effects; for total and indirect effects, see Table 3. Stump diameter and height were included as independent variables for each species but are not illustrated here. Regressions performed with ln-transformed values for stump diameter and stump height and with ln(*x* + 1)-transformed values for number of *P. grossa* and *Monochamus sutor* emergence holes per stump, and number of *Fomitopsis pinicola* fruiting bodies per stump. Untransformed values for *Trichaptum abietinum* frequency and area of bark colonized by *H. palliatus.* U = unexplained variance (1 − R^2^). *P<0·05, ***P<0·001. Goodness-of-fit index (GFI) 0·98, χ^2^=23, *P*<0·0001.

Overall, the inclusion of data on preceding species decreased the unexplained variation in *P. grossa* numbers from 96% (only stump diameter and height as predictors) to 62%. All four species listed had significant total effects on *P. grossa* in the path analysis (total effect > 0·15, *P* < 0·01, Table 3). *Fomitopsis pinicola* had only direct effects on *P. grossa*, *M. sutor* acted both directly on *P. grossa* and indirectly via *F. pinicola,* and *T. abietinum* and *H. palliatus* acted mainly indirectly on *P. grossa* via *F. pinicola* (Fig. 3, Table 3). *Trichaptum abietinum, M. sutor, H. palliatus*, stump diameter and stump height explained 25% of the variation in the number of *F. pinicola* fruting bodies. All three of these species had mainly direct effects on *F. pinicola*. Stump diameter and height explained 7% of the variation in *T. abietinum* frequency and 2% of the variation in bark area covered by *H. palliatus* (Fig. 3). Stump diameter and height explained 0% and 2% of the variation in *M. sutor, F. pinicola* numbers, respectively (data not shown).

**Table 3:** Total, direct and indirect effects of the suggested pathways between species and the effect of stump height and diameter on each of the species *Hylurgops palliatus, Monochamus sutor, Fomitopsis pinicola, Trichaptum abietinum* and *Peltis grossa* (see also Fig. 3)

		Causal effects		
			
Paths	Total effect	Direct	Indirect	*P*-value (direct)
*H. palliatus → M. sutor*	−0·28	−0·28	0	<0·0001
*H. palliatus → F. pinicola*	0·26	0·20	0·06	0·0002
*H. palliatus → Peltis grossa*	0·25	0·09	0·16	0·08
*M. sutor → F. pinicola*	−0·20	−0·20	0	0·0001
*M. sutor → P. grossa*	−0·22	−0·12	−0·10	0·02
*T. abietinum → F. pinicola*	−0·29	−0·29	0	<0·0001
*T. abietinum → P. grossa*	−0·17	−0·03	−0·14	0·4
*F. pinicola → P. grossa*	0·47	0·47	0	<0·0001
Diameter →*H. palliatus*	0·06	0·06	0	0·3
Height →*H. palliatus*	−0·11	−0·11	0	0·06
Diameter →*F. pinicola*	0·10	0·08	0·02	0·08
Height →*F. pinicola*	0·11	0·23	−0·11	<0·0001
Diameter →*T. abietinum*	−0·08	−0·08	0·01	0·1
Height →*T. abietinum*	0·27	0·27	0·03	<0·0001
Diameter →*M. sutor*	−0·03	−0·05	0·02	0·3
Height →*M. sutor*	0·08	0·05	0·03	0·4
Diameter →*P. grossa*	0·19	0·14	0·06	0·003
Height →*P. grossa*	0·09	0·07	0·03	0·2

As hypothesized, there was a negative correlation between number of emergence holes by *M. sutor* and the percentage of remaining bark in year 10 (*r* = −0·35; *P* < 0·001) and a positive correlation between the percentage of remaining bark and number of *F. pinicola* fruiting bodies in year 10 (*r* = 0·42; *P* < 0·001). The mean (±SD) number of emergence holes of *P. grossa* on stumps with and without *M. sutor* was 1·8 ± 5·6 and 6·5 ± 11, respectively. Corresponding values for stumps with and without *F. pinicola* were 5·6 ± 11 and 0·1 ± 0·5, respectively. These correlation coefficients and mean values are based on 318 stumps, i.e. site D excluded.

## Discussion

The present study gives new insight into why beetle and fungus communities in dead trees develop in different ways during wood decay. Much of the attention to whether communities are formed by stochastic or deterministic processes has been theoretical and deals with the question whether communities in similar environments may diverge towards alternative stable states (see reviews by Beisner, Haydon & Cuddington 2003; Schröder, Persson & De Roos 2005; Verhoef & Olff 2010). Empirical studies, of which only a few were conducted outside the laboratory, indicate that variable assembly history leads to divergent community assembly (Schröder, Persson & De Roos 2005 and references therein; Ejrnæs, Bruun & Graae 2006; Olito & Fukami 2009; Fukami *et al.* 2007, 2010; Chase 2003, 2010). This is in agreement with our main finding that the first-year colonizers, *H. palliatus* and *M. sutor*, affected most of the later-arriving species, and that community assembly was dependent on which of the two species that gained early dominance. Which stumps that were colonized by *H. palliatus* or *M. sutor* seems to be highly random, as no stump variable could explain their abundance, but after the colonization of these early species, we can verify two pathways of succession from early to late successional stages: facilitation; *H. palliatus*→ *P. grossa* and inhibition; *M. sutor* →*P. grossa* (Fig. 3). In the classic paper on conceptual models for ecological succession, Connell & Slatyer (1977) predicted that their ‘facilitation model’ would apply to heterotrophic succession. This has been supported by studies on wood (Renvall 1995) and dung (Slade *et al.* 2007). Our results indicate that also the ‘inhibition model’ may be applicable for succession in wood, which also has support in a recent experimental study with wood-decaying fungi (Fukami *et al.* 2010).

Another long-term study (Chase 2010), on experimental ponds, also indicated that stochastic priority effects may have long-lasting impact on community structure. Both ours and Chase's study were field studies that spanned over many years, during which time species inventories were repeatedly made on the same objects. In neither study, would an analysis based only on the final state have produced the data necessary for the conclusions made. For instance, in the present study, it would not have been possible to accurately assess the timely arrival of different species based only on stump inspection year 15, nor to determine the bark area covered by galleries of the different bark beetle species so long time after their colonization. An alternative set-up with a space-for-time substitution, i.e. a snapshot assessment of stumps of different age, could not have lead to any conclusions regarding priority effects as stumps would differ in colonization history. This demonstrates the advantage of long-term studies over snapshot or chronosequence studies when studying ecological succession in natural ecosystems, as recently highlighted by Johnson & Miyanishi (2008) and Walker *et al.* (2010).

Experimental short-term (weeks to months) studies on community assembly in microcosms (e.g. Ejrnæs, Bruun & Graae 2006; Fukami *et al.* 2007, 2010; Olito & Fukami 2009) have advantages but also limitations compared to our correlative field study. One of their main advantages is that all species can be added in all treatments, and the priority effect of each species can be systematically tested. In our study, we can only assume that all species had the opportunity to colonize the stumps. The fact that all species colonized several stumps at all sites (except D, which was excluded) indicates that this assumption holds true. An advantage of our study is that all colonizations were natural with regards to order and timing of species arrivals. Fukami *et al.* (2010) showed convincingly in a controlled experiment on wood discs that species richness of a fungal community is highly dependent on which species that was introduced first. Although the authors discuss their results in a context of natural colonization, they conclude that it is unclear to which extent the order of species occurs in the natural system. Another advantage with controlled experiments over our study is that the environment in which all interactions take place is homogeneous, yielding low statistical variance and enabling powerful hypothesis testing. On the other hand, heterogeneity is a major feature of natural ecosystems and important to consider when estimating the strength of species interactions (Hunter & Price 1992). The goal of our study was to test the hypothesis of long-term priority effects in a natural ecosystem and obviously the strength of these effects were large enough to appear also in the presence of variance caused by variables beyond our control.

Our study tests a hypothesis of causal relationships using path analysis, which is based on correlations. But positive or negative correlations between two species may be driven by similar or dissimilar habitat requirements, indicating a possible lack of causality in the association (Ovaskainen, Hottola & Siitonen 2010). Our data on correlations between stump variables and species do not indicate that similar or dissimilar habitat requirements were a cause for significant correlation between two species. For instance, stump height had a positive effect on both *F. pinicola* and *T. abietinum* (Table 3), but the correlation between these two species was negative. Also, bark cover (not included in the analysis) was positively correlated with both these species. *Peltis grossa* was positively correlated with diameter, but the four preceding species, all significantly correlated with *P. grossa*, were not correlated with diameter. Correlations should nevertheless be interpreted with caution and therefore it is important that suggested relationships in a path analysis reflect rational causality, and that these hypothetical causal relationships be established before analysing data (Mitchell 1993; Petraitis, Dunham & Niewiarowski 1996). Both of these requirements were met in the present study.

The two fungus species *T. abietinum* and *F. pinicola* only occasionally occurred on the same stump but were then always spatially separated. These results are largely in accordance with those from another field study on high stumps (Abrahamsson, Lindbladh & Rönnberg 2008). Ovaskainen, Hottola & Siitonen (2010) found that *T. abietinum* and *F. pinicola* often occurred together on the same logs, and they hypothesized that this was either due to higher competitive ability of *T. abietinum*, which made it possible to colonize logs already occupied by *F. pinicola*, or due to niche separation between the two species. We could not see any signs of niche separation in the present study, as either species could be found anywhere on a stump and the abundance of their fruiting bodies was negatively correlated. Instead, our results and observations suggest that competition occurred between *T. abietinum* and *F. pinicola*. In a laboratory study, Holmer, Renvall & Stenlid (1997) found that *T. abietinum* was the superior competitor of the two fungus species when introduced simultaneously and even so in combinations where *T. abietinum* was introduced in lower dose than *F. pinicola*. Thus, when colonizing dead wood objects simultaneously, *T. abietinum* is likely to reduce the potential substrate available to *F. pinicola.*

The final model differed from the hypothetical model in several aspects. First, the two bark beetle species *I. typographus* and *P. chalcographus* had no effect on *P. grossa* numbers. Second, the hypothesis that *H. palliatus* had an indirect effect on *F. pinicola* via *M. sutor* was rejected. Instead, almost all of its effect was direct. Third, there was an unpredicted direct effect of *M. sutor* on *P. grossa*. The direct positive association between *H. palliatus* and *F. pinicola* might be explained if *H. palliatus* carried *F. pinicola* spores or mycelia. Several bark beetle species that fly during *F. pinicola* sporulation have been shown to become contaminated with spores to a high degree during their flight (Pettey & Shaw 1986). Also, colonization by wind borne spores may have been facilitated by beetle holes in the bark. The reason why *I. typographus* and *P. chalcographus* did not facilitate *F. pinicola* colonization may be a result of differences in the flight periods of the bark beetles and in the sporulation periods of the fungi. Local meteorological data indicated that the interval between the main flights of *H. palliatus* with a flight threshold of 15°C, and of *I. typographus* and *P. chalcographus*, each with a flight threshold of 20°C, would have exceeded 6 weeks in both 1994 and 1995. In Scandinavia, *F. pinicola* sporulation starts in early spring, just after snow-melt (Hågvar 1999), which coincides with the main flight of *H. palliatus*. In contrast, *T. abietinum* sporulates later in the summer as a result of having annual fruiting bodies. Thus, it may be that during the colonization of the stumps by *I. typographus* and *P. chalcographus*, *F. pinicola* had to compete with the superior competitor *T. abietinum*. The unpredicted direct effect of *M. sutor* on *P. grossa* is not easily explained but could be due to *M. sutor* larvae feeding deep within the sapwood and reducing habitat quality there for *P. grossa*.

Studies on beetle communities in dead trees have a long tradition, especially in Fennoscandia. The works by Saalas (1917) and Palm (1951, 1959), in which detailed descriptions of beetle communities in various stages of wood decay are provided, deserve special mentioning. However, earlier studies on community ecology of wood-living beetles have not been strongly linked to succession or assembly theories. As such, this study is a novel contribution. Our study also exemplifies new options for species-oriented conservation measures. In the case of *H. palliatus*, *M. sutor* and *P. grossa,* cutting of stumps during fall and winter (after the flight of *M. sutor* and before the flight of *H. palliatus*) would be a good option to increase the probability for subsequent colonization by *P. grossa*, whereas cutting during spring and summer (after the flight of *H. palliatus* and before the flight of *M. sutor*) would have the opposite effect. Thus, exploring links between early, well-understood species guilds and later, more poorly understood species guilds may offer new possibilities for managing populations of threatened species.

## References

[b1] Abrahamsson M, Lindbladh M, Rönnberg J (2008). Influence of butt rot on beetle diversity in artificially created high-stumps of Norway spruce. Forest Ecology and Management.

[b2] Alford RA, Wilbur HM (1985). Priority effects in experimental pond communities, competition between Bufo and Rana. Ecology.

[b3] Almany GR (2003). Priority effects in coral reef fish communities. Ecology.

[b4] Beisner BE, Haydon DT, Cuddington K (2003). Alternative stable states in ecology. Frontiers in Ecology and the Environment.

[b5] Boddy L (2000). Interspecific combative interactions between wood-decaying basidiomycetes. FEMS Microbiology Ecology.

[b6] Boddy L (2001). Fungal community ecology and wood decomposition processes in angiosperms, from standing tree to complete decay of coarse woody debris. Ecological Bulletins.

[b7] Boddy L, Jones TH, Boddy L, Frankland JC, vanWest P (2008). Interactions between Basidiomycota and Invertebrates. Ecology of Saprotrophic Basidiomycetes.

[b8] Chase JM (2003). Community assembly, when does history matter?. Oecologia.

[b9] Chase JM (2010). Stochastic community assembly causes higher biodiversity in more productive environments. Science.

[b10] Connell JH, Slatyer RO (1977). Mechanisms of succession in natural communities and their role in community stability and organization. The American Naturalist.

[b11] Dahlberg A, Stokland JN (2004). *Vedlevande arters krav på substrat - sammanställning och analys av 3600 arter. [Wood living species substrate preferences – an analysis of 3600 species]* (in Swedish with English summary).

[b12] Diamond JM, Cody ML, Diamond JM (1975). Assembly of species communities. Ecology and Evolution of Communities.

[b13] Drake JA (1991). Community assembly mechanics and the structure of an experimental species ensemble. American Naturalist.

[b14] Ehmann WJ, MacMahon JA (1996). Initial tests for priority effects among spiders that co-occur on sagebrush shrubs. Journal of Arachnology.

[b15] Ehnström B (2001). Leaving Dead Wood for Insects in Boreal Forests - Suggestions for the Future. Scandinavian Journal of Forest Research.

[b16] Ehnström B, Axelsson R (2002). *Insektsgnag i bark och ved*. (in Swedish). SLU.

[b17] Eidmann HH, Klingström A (1990). Skadegörare i skogen [Pests of the forest]. (in Swedish).

[b18] Ejrnæs R, Bruun HH, Graae BJ (2006). Community assembly in experimental grassland, suitable environment or timely arrival?. Ecology.

[b19] Fjellberg A, Hansen SO (1997). Peltis grossa (L.) still present in Norway (Coleoptera, Trogossitidae). Faunan Norwegica Serie B.

[b20] Forestry Compendium (2005). CD-ROM Edition. CAB International. http://www.cabicompendium.org/fc.

[b21] Fukami T, Beaumont HJE, Zhang X-X, Rainey PB (2007). Immigration history controls diversification in experimental adaptive radiation. Nature.

[b22] Fukami T, Dickie IA, Wilkie JP, Paulus BC, Park D, Roberts A, Buchanan PK, Allen RB (2010). Assembly history dictates ecosystem functioning, evidence from wood decomposer communities. Ecology Letters.

[b23] Gärdenfors U (2010). The 2010 redlist of Swedish species.

[b24] Hågvar S (1999). Saproxylic beetles visiting living sporocarps of *Fomitopsis pinicola* and *Fomes fomentarius. Norw*. Journal Entomology.

[b25] Holmer L, Renvall P, Stenlid J (1997). Selective replacement between species of wood-rotting basidiomycetes, a laboratory study. Mycological research.

[b26] Hubbel SP (2001). The Unified Neutral Theory of Biodiversity and Biogeography.

[b27] Hunter MD, Price PW (1992). Playing chuted and ladders: heterogeneity and relative roles of bottom-up and top-down forces in natural communities. Ecology.

[b28] Johnson EA, Miyanishi K (2008). Testing the assumptions of chronosequences in succession. Ecology Letters.

[b29] Jonsell M, Schroeder M, Weslien J (2005). Saproxylic beetles in high stumps of spruce -fungal flora important for determining the species composition. Scandinavian Journal of Forest Research.

[b30] Jöreskog KG, Sörbom D (1985). LISREL VI; Analysis of Linear Structural Relationships by Maximum Likelihood, Instrumental Variables, and Least Squares.

[b31] Käärik A, Rennerfelt E (1957). Investigations on the fungal flora of spruce and pine stumps. Meddelanden från Statens Skogsforskningsinstitut.

[b32] Kennedy PG, Bruns TD (2005). Priority effects determine the outcome of ectomycorrhizal competition between two Rhizopogon species colonizing Pinus muricata seedlings. New Phytologist.

[b33] Krasutskii BV (2007). Coleoptera associated with *Fomitopsis pinicola* (Sw., Fr.) Karst.(Basidiomycetes, Aphyllophorales) in the forests of the Urals and Transurals. Entomological Reveiw.

[b34] Lindhe A, Åsenblad N, Toresson HG (2004). Cut logs and high stumps of spruce, birch, aspen and oak – nine years of saproxylic fungi succession. Biological conservation.

[b35] Lortie J, Brooker RW, Choler P, Kikvidze Z, Michalet R, Pugnaire FJ, Callaway RM (2004). Rethinking plant community theory. Oikos.

[b36] Mitchell RJ, Scheiner SM, Gurevitch J (1993). Path analysis, pollination. Design and Analysis of Ecological Experiments.

[b37] Nee S, Stone G (2003). The end of the beginning for neutral theory. Trends in Ecology and Evolution.

[b38] Nuss I (1986). Zur Ökologie der Porlinge. II. Die Entwicklungsmorphologie der Fruchtkörper und ihre Beeinflussung durch klimatische und andere Faktoren.

[b39] Olito C, Fukami T (2009). Long-term effects of predator arrival timing on prey community succession. American Naturalist.

[b40] Ovaskainen O, Hottola J, Siitonen J (2010). Modeling species co-occurrence by multivariate logistic regression generates new hypotheses on fungal interactions. Ecology.

[b41] Palm T (1951). Die Holz- und Rindenkäfer der nordschwedischen Laubbäume. Meddelanden från Statens Skogsforskningsinstitut.

[b42] Palm T (1959). Die Holz- und Rindenkäfer der süd- und mittelschwedischen Laubbäume. Opuscula Entomologica Supplementum.

[b43] Persson Y, Ihrmark K, Stenlid J (2011). Do bark beetles facilitate the establishment of rot fungi in Norway spruce?. Fungal Ecology.

[b44] Persson Y, Vasaitis R, Långström B, Öhrn P, Ihrmark K, Stenlid J (2009). Fungi vectored by bark beetle *Ips typographus* following hibernation under the bark of standing trees and in the forest litter. Microbial Ecology.

[b45] Petraitis PS, Dunham AE, Niewiarowski PH (1996). Inferring multiple causality: the limitations of path analysis. Functional Ecology.

[b46] Pettey TM, Shaw CG (1986). Isolation of *Fomitopsis pinicola* from in-flight bark beetles (Coleoptera: Scolitdae). Canadian Journal of Botany.

[b47] Rayner ADM, Boddy L (1988). Fungal Decomposition of Wood: Its Biology and Ecology.

[b48] Renvall P (1995). Community structure and dynamics of wood-rotting Basidiomycetes on decomposing conifer trunks in northern Finland. Karstenia.

[b49] Saalas U (1917). Die Fichtenkäfer Finnlands I. Annales Academiae Scientarium Fennicae Ser. A..

[b50] SAS-inst. (2003). SAS for Windows ver. 9.1.3. SAS Institute Inc.

[b51] Schröder A, Persson L, De Roos AM (2005). Direct experimental evidence for alternative stable states: a review. Oikos.

[b52] Schroeder LM, Weslien J (1994). Interactions between the phloem-feeding species *Tomicus piniperda* (Col, Scolytidae) and *Acanthocinus aedilis* (Col, Cerambycidae), and the predator *Thanasimus formicarius* (Col, Cleridae) with special reference to brood production. Entomophaga.

[b53] Schroeder LM, Weslien J, Lindelöw Å, Lindhe A (1999). Attacks by bark- and wood-boring Coleoptera on mechanically created high stumps of Norway spruce in the two years following cutting. Forest Ecology and Management.

[b54] Shorrocks B, Bingley M (1994). Priority effects and species coexistence: experiments with fungal-breeding Drosophila. Journal of Animal Ecology.

[b55] Shulman MJ, Ogden JC, Ebersole JP, McFarland WN, Miller SL, Wolf NG (1983). Priority effects in the recruitment of juvenile coral-reef fishes. Ecology.

[b56] Slade EM, Mann DJ, Villanueva JF, Lewis OT (2007). Experimental evidence for the effects of dung beetle functional group richness and composition on ecosystem function in a tropical forest. Journal of Animal Ecology.

[b57] Swenson NG, Anglada-Cordero P, Barone JA (2011). Deterministic tropical tree community turnover: evidence from patterns of functional beta diversity along an elevational gradient. Proceedings of the Royal Society.

[b58] Swift MJ (1977). The ecology of wood decomposition. Science progress.

[b59] Tikkanen O-P, Martikainen P, Hyvärinen E, Junninen K, Kouki J (2006). Red-listed boreal forest species of Finland: associations with forest structure, tree species, and decaying wood. Annales Zoologici Fennici.

[b60] Verhoef HA, Olff H, Verhoef HA, Morin PJ (2010). Trophic dynamics of communities. Community Ecology. Processes, Models and Applications.

[b61] Viktorsson J (2009). Digital comprehensive summaries of Uppsala Dissertations from the Faculty of Science and Technology. Community Assembly and Spatial Ecology of Saproxylic Coloeoptera.

[b62] Walker LR, Wardle DA, Bardgett RD, Clarlson BD (2010). The use of chronosequences in studies of ecological succession and soil development. Journal of Ecology.

[b63] Warner SC, Dunson WA, Travis J (1991). Interaction of Ph, Density, and Priority Effects on the Survivorship and Growth of 2 Species of Hylid Tadpoles. Oecologia.

[b64] Wilding N, Collins M, Hammond PM, Webber JF (1989). *Insect-Fungus Interactions*, 14th Symposium of the Royal Entomological Society of London in Collaboration with the British Mycological Society.

